# A living document: reincarnating the research article

**DOI:** 10.1186/s13063-015-0666-5

**Published:** 2015-04-11

**Authors:** Daniel R Shanahan

**Affiliations:** BioMed Central, 236 Gray’s Inn Road, London, WC1X 8HB UK

## Abstract

The limitations of the traditional research paper are well known and widely discussed; however, rather than seeking solutions to the problems created by this model of publication, it is time to do away with a print era anachronism and design a new model of publication, with modern technology embedded at its heart. Instead of the current system with multiple publications, across multiple journals, publication could move towards a single, evolving document that begins with trial registration and then extends to include the full protocol and results as they become available, underpinned by the raw clinical data and all code used to obtain the result. This model would lead to research being evaluated prospectively, based on its hypothesis and methodology as stated in the study protocol, and move away from considering serendipitous results to be synonymous with quality, while also presenting readers with the opportunity to reliably evaluate bias or selective reporting in the published literature.

## Background

When the Royal Society first advocated the transparent and open exchange of ideas backed by experimental evidence, the Society was widely ridiculed. At the time, the concept of openly sharing your work in a research article was highly controversial. It was not uncommon for new discoveries to be announced by describing them in papers coded in anagrams or cyphers [1] - reserving priority for the discoverer, but largely indecipherable for anyone not already in on the secret. Both Newton and Leibniz used this device.

As you might imagine, this led to a number of disputes over priority, and it seems rather absurd to us today. However, since the advent of the research article over 300 years ago, academic publishing has been viewed as a way of minuting what was done and sharing the results [2].

Three-hundred years is a long time; technology has seen huge advancements over the last 20 years alone. The Internet has seismically disrupted the way we both communicate and find data, displacing traditional information delivery and becoming an integral part of life for millions. The increased availability of information has led to calls for greater transparency in research - for a clear, detailed record of exactly what was done, and how, to allow the work to be reliably reproduced. Despite this, many journals perpetuate the view of research articles as ‘minutes’. Print era anachronisms persist through the continuation of page and word limits and the release of discrete issues, as if all articles remain subject to print-only production constraints. Indeed, it was only recently that certain top journals elected to remove the word limits on their methods sections [3]. It brings to mind Fermat’s aside to his infamous last theorem written in *Artimetica* in 1637, claiming that the proof for what he stated was ‘too large to fit in the margin’ [4].

### Where is the value in the research article?

Research only has value if the methods used are appropriate and it is reproducible [5]. However, in modern biomedical research, the majority of published research claims may in fact be impossible to reproduce [6-8]. Many reported results are later refuted, and controversy is seen across the entire range of research designs, from randomised controlled trials (RCTs) to traditional epidemiological studies [9-11]. Even for studies following ‘gold standard’ reporting and open data policies, researchers face difficulties in replicating them [12].

One possible explanation for this, as hypothesized by Ioannidis *et al*., is that controversial data are attractive to investigators and editors, making contradictory results more likely to be published than confirmatory ones [7,13]. However, reviews of published trials consistently show that, even for those articles that are published, key information is frequently missing [14]. There is also growing evidence that space pressures influence the way that researchers choose to write up their studies, with a bias in favour of selecting those outcomes and analyses that are statistically significant [15,16].

It is concerns like these that led to widespread calls for registering trials [17,18], pre-specifying the research outcomes and methods. Similarly, reporting guidelines were created to outline the minimum information required for a full and complete report, with evidence that the adoption of reporting guidelines, such as the CONSORT Statement, has led to improved reporting [19]. Journals like *Trials* also encourage prospective publication of study protocols, which had rarely been possible in paper-based journals [20,21]; publication of study protocols allows for more detailed discussion of methodological issues, which can be referenced when reporting the main trial results [22].

However, researchers need access to all of the relevant information, to reliably evaluate bias or selective reporting in clinical trials. As any systematic reviewer can tell you, identifying all publications related to a single clinical trial can be a Sisyphean task. Indeed, there are initiatives in the works to assist with this effort [23], but regardless of the success of these initiatives, this simply serves to highlight the absurdity of having separate ‘protocol’ papers and ‘results’ papers. These are all solutions to a problem that we ourselves have created.

Indeed in 1963, Peter Medawar asked whether the scientific paper itself was a fraud. He maintained that the research article was a ‘travesty […] which editors themselves often insist upon’, insisting that research articles give ‘a totally misleading narrative of the processes of thought that go into the making of scientific discoveries’. A paper’s fraud, Medawar argued, lay mainly in its form [24].

## Main text

### A ‘living’ document

It is time to ask ourselves whether the research article itself has now become an anachronism. In contrast to an article of the print era, an article that has been published online is not a sealed black box. It can be updated, amended, extended and indeed directly linked to other articles and data.

So why do we with persist with this paradigm whereby each new ‘stage’ in the research cycle results in a separate publication? It is time for the research article to move beyond the now-obsolete print model and truly embrace the freedom that online publication gives us, moving towards living documents, with a single article for a single piece of research.

**Figure 1:**
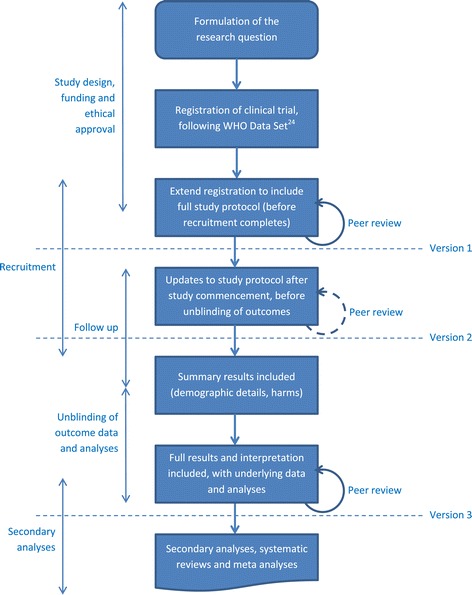
Workflow for a living document of a randomized controlled trial.

It is a powerful concept. Currently, a single clinical trial can result in a study protocol and traditional results paper (or papers), as well as commentaries, secondary analyses and, eventually, systematic reviews, among others [25]. Instead of multiple publications, across multiple journals, with associated different publishing formats, researchers could register our intention to perform a clinical trial, detailing the standard 20-items that are currently required [26]. This could then be extended to the full study protocol, building on the skeleton that was provided on registration. Once they have completed the study, they can then update the document to include the results and analyses performed, without having to rewrite the methods and risk self-plagiarism (Figure 1).

While the article would evolve over time, substantive additions to the article that were judged to impact the scientific validity of the literature would require peer review, as shown in Figure 1. In these cases, the article could be frozen into a discrete version, with the reviewer reports associated with it. This model is already used by journals that operate on a post-publication peer review process, such as *F1000Research* and *ScienceOpen* [27,28]. Citations to the document would then be required to include the access date, which would uniquely identify the version of the article referred to.

Creation of a living document that could be updated as required, would allow researchers to capture the information in real time, allowing for simpler concurrent research projects and facilitating reporting, as the authors would only need to focus on a specific section at any one time, rather than attempt to identify and follow all the relevant reporting guidelines for the study from over two hundred [29], when finally writing it up.

This concept of an evolving document is already demonstrated for systematic reviews by the *Living Reviews* series of open access journals, which allow the authors to regularly update their articles to incorporate the latest developments in the field [30]; however, it has not been applied to primary research. Extending this concept to primary research could cause the article to become unwieldy under the traditional IMRAD headings, particularly for large clinical trials with an associated large number of analyses; however, this is already the case for traditional results papers. These concerns have led to journals requiring core statistical methods to be included in the figure captions of presented results, as well as innovative navigation tools to allow readers to view the research methods and analyses simultaneously, for example, eLife Lens [31].

Reproducibility also requires the ability to manipulate and re-analyse data; therefore, as stated by Claerbout, in addition to any summary results included to support the written interpretation, the document should link to the raw clinical data and all code used to obtain the result [32]. An immense amount of work has gone into the creation of reproducible research platforms and the concept of ‘literate programming’. This has led to the development of a whole programming format, SWeave, which allows the creation of dynamic reports with code integrated into LaTeX documents, which can be updated automatically if data or analyses change [33]. Similarly, Kauppinen *et al*. established and defined *Linked Open Science*, an approach to interconnect scientific assets to enable transparent, reproducible and transdisciplinary research [34].

The dramatic decrease in data storage costs [35] and emergence of virtual environments, such as Arvados [36], make it possible to enable reproducibility of data analysis with versioned scripts and tools. Trialists can deposit the data, tools and scripts they used to analyse the data, allowing readers to see how robust the visualisations and statistics embedded in the paper are.

### Limitations

Underpinning the results and interpretations with the original data and analyses tools has obvious benefits for conducting meta-analyses and systematic reviews, as well as for reproducibility of research. Similarly, creation of an evolving document for a single research project would make evaluation of selective reporting of both analyses and outcomes straightforward, as all the necessary information and methods would be reported in the same place. However, there are limitations compared with the existing publication paradigm. As the article is able to continuously evolve, there is no permanent ‘version of record’; therefore, the articles would need ongoing curation, which could cause issues in the event of a journal closure. As stated by Barnes in the *Science Code Manifesto*, ‘Source code must remain available, linked to related materials, for the useful lifetime of the publication’ [37]. While a discrete version could be created in such instances, it would prevent further updating of the article, which could lead to the literature being incomplete.

Furthermore, by encouraging and facilitating reproduction, this raises the issue of how to combine original research articles with follow-up replication or analyses by a different group of authors. Including these follow-up studies in the original living document could cause issues with accreditation; however, it could also help to emphasise that reproduction is a fundamental part of research, leading to large research consortia, as currently seen in physics and genetics. An alternative to this would be to adapt the existing ‘update’ article types, creating a separate citation, but accessed in tandem to the original article.

A continuously-evolving document would also undermine existing methods of evaluating the impact of a piece of work, particularly metrics like the Impact Factor or any article- or journal-level metric that relies on the date of publication. As study protocols are seldom cited, a living document is unlikely to be cited regularly until the article has been expanded to include the results and interpretation; however, this means that citations to the article could come a number of years after original publication and, therefore, would not be included in the Impact Factor calculations. However, this could also prove an advantage, as implementation of living documents, as described above, would require a journal to commit to publishing the results of a piece of research based on the methodological quality of the protocol, regardless of outcome or significance of findings, or considered level of interest. This could help to move away from a results focus to considerations of the question asked and the processes used, when evaluating scientific validity.

Current technology means that this form of publication is theoretically possible already. However, contemporary cultural attitudes and workflows, within both publishing and academia, along with research conduct and evaluation, present barriers to its implementation. Evaluating research prospectively, based on its hypothesis and methodology as stated in the study protocol, and then continuously updating the article as results and data become available, moves us past considering serendipitous results as being synonymous with quality, while also giving us the opportunity to reliably evaluate bias or selective reporting in the published literature.

## Conclusion

The current incarnation of the research article has persisted for over 300 years; however, evolving technology makes it, not simply anachronistic, but effectively fraudulent. While cultural attitudes and establishments remain a large hurdle, both within the publishing and academic communities, the ongoing drive towards transparency and reproducibility make it no longer acceptable to continue to perpetuate a centuries-old absurdity.

## Abbreviations

CONSORT: Consolidated Standards of Reporting Trials
IMRAD: Introduction, Methods, Results and Discussion
